# Therapeutic intensity and patient-reported outcomes among heart failure patients in a pharmacist-led medication therapy adherence clinic program in Malaysia

**DOI:** 10.3389/frhs.2026.1784432

**Published:** 2026-04-13

**Authors:** Abdullah Faiz Zaihan, Shairyzah Ahmad Hisham, Nurul Ameera Huda Mohamad Hafiz, Nur Irdina Athilah Khairul Hazamy, Aisyah Zahraa Termizy, Nurul Izzah Roslan, Perishithaa M. Ganesan, Noor Salihah Yahaya, Zhiyun Wong, Muhammad Iqbal Mohd Arqam, Chia Siang Kow

**Affiliations:** 1 Department of Pharmacy, Shah Alam Hospital, Shah Alam, Selangor, Malaysia; 2 School of Pharmacy, University of Nottingham Malaysia, Semenyih, Selangor, Malaysia; 3 School of Pharmacy, IMU University, Kuala Lumpur, Malaysia; 4 Faculty of Pharmacy, University of Cyberjaya, Cyberjaya, Selangor, Malaysia; 5 School of Applied Sciences, University of Huddersfield, Huddersfield, United Kingdom; 6 School of Pharmacy, Faculty of Medical and Life Sciences, Sunway University, Petaling Jaya, Selangor, Malaysia

**Keywords:** canagliflozin, clinical outcomes, ertugliflozin, medication compliance, pharmacist

## Abstract

**Introduction:**

Guideline-directed medical therapy (GDMT) and newer agents such as sodium-glucose cotransporter 2 inhibitors (SGLT2i) have significantly improved outcomes in heart failure (HF). However, their impact on patient experience—including medication adherence, treatment burden, and quality of life (QOL)—remains less understood in Malaysia, especially in structured outpatient settings such as Medication Therapy Adherence Clinic (MTAC).

**Aim:**

To evaluate associations between therapeutic intensity (full GDMT, SGLT2i use, and medication load) and patient-reported outcomes (PROs) in HF patients enrolled in an MTAC program.

**Method:**

A cross-sectional study was conducted among 78 HF patients at a secondary hospital MTAC in Shah Alam, Malaysia. PROs were assessed using the Malaysia Medication Adherence Assessment Tool (MyMAAT-12), the Treatment Burden Questionnaire (TBQ), and WHOQOL-BREF. Full GDMT was defined as concurrent use of a RAAS inhibitor, beta blocker, MRA, and SGLT2i. Group comparisons were performed using Mann–Whitney U tests, and associations between medication load and PROs were assessed using Spearman correlation.

**Results:**

The mean age of patients was 57.3 ± 11.5 years; 74.4% were male. Most were on polypharmacy (mean medication classes: 6.6 ± 1.2); 64.1% were on full GDMT and 82.1% were prescribed SGLT2i. Median adherence, burden, and QOL scores were 47.0 (IQR: 40.0–53.0), 39.0 (IQR: 24.0–58.0), and 88.0 (IQR: 77.0–98.0), respectively. Patients on full GDMT or ARNI had similar adherence, burden, and QOL scores compared to those not on these therapies. However, SGLT2i users reported significantly lower adherence (*p* = 0.037) and QOL (*p* = 0.018). Medication load was positively correlated with QOL (*r* = 0.295, *p* = 0.009), but not with adherence or burden.

**Conclusion:**

In this MTAC-supported cohort, polypharmacy and full GDMT were not associated with increased burden or reduced adherence. A modest positive association between medication count and QOL was observed. SGLT2i use was associated with lower adherence and QOL, although these findings are exploratory and may reflect differences in disease severity or treatment complexity rather than a direct effect of therapy. Overall, these results provide observational insights into the relationship between therapeutic intensity and PROs within a pharmacist-led care setting, and warrant confirmation in larger, longitudinal studies with appropriate adjustment for clinical variables.

## Introduction

Heart failure (HF) remains a major global health challenge, with rising prevalence, frequent hospitalizations, and substantial socioeconomic burden. Despite advances in pharmacotherapy and care delivery, patients with HF often experience high symptom burden, complex therapeutic regimens, and diminished quality of life (QOL) (1). Guideline-directed medical therapy (GDMT)—including renin-angiotensin-aldosterone system (RAAS) inhibitors, beta blockers, mineralocorticoid receptor antagonists (MRA), and most recently, sodium-glucose cotransporter 2 inhibitors (SGLT2i)—has been shown to reduce morbidity and mortality in HF with reduced ejection fraction (HFrEF) (2–9). However, translating these evidence-based recommendations into meaningful improvements in patient experience remains a clinical challenge.

While clinical trials consistently demonstrate the prognostic benefit of GDMT, real-world implementation is often complicated by polypharmacy, comorbidities, healthcare access, and patient preferences. The expansion of HF pharmacotherapy has led to increased medication load, which may affect treatment adherence and perceived burden. Notably, while polypharmacy is often associated with negative outcomes in older adults (10), in HF management, a higher number of medication classes may also reflect comprehensive, guideline-concordant care. Understanding how patients perceive and cope with the intensity of treatment—particularly in structured programs such as Medication Therapy Adherence Clinics (MTACs)—is essential for optimizing both clinical outcomes and patient-reported measures (11, 12).

SGLT2 inhibitors have emerged as a cornerstone of HF therapy with demonstrated cardiovascular and renal benefits (13, 14). However, their real-world impact on patient-centered outcomes such as QOL and adherence remains less well defined, especially in low- and middle-income countries. Factors such as side effects, medication complexity, cost, and health literacy may influence how patients perceive and adhere to SGLT2i therapy. Although these agents are well tolerated in most clinical trial populations, their introduction into polypharmacy-heavy regimens may have unintended effects on patient engagement, satisfaction, and adherence.

In Malaysia, MTACs provide pharmacist-led, patient-centered services aimed at optimizing medication use, improving disease knowledge, and supporting adherence (15). While the MTAC model has shown promise in chronic disease management, data on its role in supporting complex HF pharmacotherapy and how it impacts patient-reported outcomes in this population are limited.

## Aim

To address these gaps, this study aimed to examine the association between therapeutic intensity and patient-reported outcomes—including QOL, treatment burden, and medication adherence—among HF patients receiving care in a Malaysian MTAC. Specifically, we investigated whether full GDMT, total number of medication classes (as a proxy for treatment complexity), and SGLT2i use influenced patient perceptions and behaviors in a real-world setting. By capturing both therapeutic structure and patient experience, this study aimed to contribute important insights to the implementation of HF care in multidisciplinary outpatient environments.

## Ethics approval

This study was approved by the Medical Research and Ethics Committee (MREC), Ministry of Health Malaysia (NMRR ID-25-01798-K5L). The study adhered to the ethical principles outlined in the Declaration of Helsinki. Written informed consent was obtained from all participants prior to data collection. All participants consented to the use of their anonymized data for research publication purposes.

## Method

### Study design and setting

This was a single-center, cross-sectional observational study conducted at the Heart Failure Medication Therapy Adherence Clinic (HF-MTAC) at Shah Alam Hospital, a secondary public hospital under the Ministry of Health, Malaysia. The MTAC program is pharmacist-led and provides structured follow-up, medication optimization, and therapeutic counselling for patients with chronic diseases, including HF. The study aimed to evaluate the impact of therapeutic regimens on patient-reported outcomes within this structured care model. Data were collected between 1st January 2025 and 31st March 2025.

### Study population and eligibility criteria

Participants were recruited from HF-MTAC during scheduled follow-up visits. Patients were eligible if they:

1. Were ≥18 years old,
2. Had a confirmed diagnosis of HF,
3. Were actively managed within the HF-MTAC program,
4. Had been prescribed at least one medication for HF (e.g., RAAS inhibitor, beta blocker, MRA, or SGLT2i),
5. Were able to communicate in Malay or English,
6. Provided written informed consent.

Exclusion criteria included patients with significant cognitive impairment, active psychiatric illness, language barrier, or those with incomplete medication or follow-up records.

### Therapeutic regimen definitions

- Full GDMT was defined as the concurrent prescription of four major pharmacological classes recommended for HFrEF:
  - A RAAS inhibitor: ACE inhibitor, ARB, or ARNI,
  - A beta blocker,
  - A MRA, and
  - A SGLT2i.

Prescription status was confirmed using electronic medical records and MTAC pharmacist documentation.

- Medication load was calculated as the number of distinct medication classes prescribed from a list of 12 predefined categories. These included the four GDMT components, as well as loop diuretics, oral antidiabetics, insulin, statins, antiplatelets, anticoagulants, and others commonly used in HF comorbidity management.

### Assessment of patient-reported outcomes (PROs)

Patient-reported outcomes were assessed using three structured, validated instruments:

- Medication adherence was assessed using the Malaysia Medication Adherence Assessment Tool (MyMAAT-12), a 12-item self-administered questionnaire developed and validated for Malaysian patients with chronic diseases (16). The tool measures key dimensions of adherence, including forgetfulness, treatment understanding, side effect concerns, and intentional non-adherence. Responses are scored on a 5-point Likert scale (ranging from “strongly disagree” to “strongly agree”), with higher total scores indicating better adherence. A total score of 54 or above is considered indicative of adherence.
- Treatment burden was assessed using the 15-item Treatment Burden Questionnaire (TBQ) (17). The TBQ evaluates the perceived workload of healthcare management across multiple domains, including medication complexity, frequency of appointments, administrative burden, lifestyle impact, and treatment-related distress. Each item is rated on a 0–10 scale, with higher scores indicating greater perceived burden. The TBQ has been validated in chronic disease populations and adapted linguistically and contextually for the Malaysian setting.
- Quality of life (QOL) was measured using the World Health Organization Quality of Life—BREF (WHOQOL-BREF) (18). This 26-item instrument covers four domains: physical health, psychological well-being, social relationships, and environment. Each item is rated on a 5-point scale, with higher scores indicating better perceived QOL. The WHOQOL-BREF is widely validated across cultural and clinical contexts and was selected for its comprehensiveness and suitability in evaluating overall well-being in HF patients.

### Survey administration

All questionnaires were administered in person during scheduled MTAC consultations. A trained MTAC pharmacist or research assistant introduced the study, explained the purpose of the survey, and guided the patient through the consent process. Patients were offered a quiet, private consultation room to complete the questionnaire.

Participants could complete the questionnaire independently or request assistance if needed. Assistance was provided in a neutral tone, with the administrator reading questions aloud and recording responses verbatim when required (e.g., due to low literacy, vision problems, or frailty). Patients were encouraged to seek clarification if they did not understand any item. Both Malay and English versions of the questionnaire were available.

The average time to complete the survey was 15–25 min. Completed forms were reviewed immediately by the administrator for missing or unclear responses, which were clarified in real time. Data were subsequently anonymized, assigned a unique study code, and entered into a password-protected Excel database.

### Data collection and clinical variables

In addition to survey responses, clinical and sociodemographic data were collected from the patients' MTAC records and verified during the clinic visit. These included:

- Age, gender, ethnicity, marital status, education level,
- Employment status,
- Monthly household income,
- Medication history,
- Co-existing comorbidities, and
- Current pharmacological regimen.

Medication class allocation was determined based on active prescriptions at the time of assessment, verified against electronic pharmacy records and patient medication reconciliation documents.

### Statistical analysis

All data were analyzed using IBM SPSS Statistics version 26.0. Descriptive statistics were used to summarize patient characteristics and outcome scores.

- Continuous variables were reported as mean ± SD or median (IQR) depending on data distribution.
- Categorical variables were summarized as frequencies and percentages.

Comparisons between groups (e.g., full GDMT vs. non-full GDMT; SGLT2i use vs. non-use) were performed using the Mann–Whitney *U*-test, given the non-parametric nature of the outcome distributions.

Associations between medication load (as a continuous variable) and each outcome domain (QOL, burden, adherence) were assessed using Spearman's rank correlation coefficient.

Non-parametric methods were selected due to the non-normal distribution of variables and the modest sample size, which limited the feasibility of more complex multivariable modelling.

A two-tailed *p*-value of <0.05 was considered statistically significant.

### Ethical considerations

This study was approved by the Medical Research and Ethics Committee (MREC), Ministry of Health Malaysia (Reference No: NMRR ID-25-01798-K5L). Written informed consent was obtained from all participants prior to data collection. All data were handled in accordance with Good Clinical Practice guidelines and ethical principles of the Declaration of Helsinki.

## Results

### Patient characteristics

A total of 78 patients with HF were included in the study. The mean age was 57.3 years (SD ±11.5), with most patients falling between the ages of 50 and 70 years. The majority were male, comprising 58 patients (74.4%). In terms of ethnicity, 60 patients (76.9%) were Malay, 11 (14.1%) were Indian, five (6.4%) were Chinese, and two (2.6%) belonged to other ethnic groups.

Employment status varied: 27 patients (34.6%) were unemployed, 23 (29.5%) were retired, 22 (28.2%) were employed, and six (7.7%) were homemakers. The median gross monthly household income was RM1450 (IQR: RM0–3000), while the mean was RM1696.8 (SD ±1676.8), reflecting a wide range of socioeconomic backgrounds. A summary of sociodemographic and clinical characteristics is provided in Table 1.

**Table 1:** Sociodemographic and clinical characteristics of included patients (*N* = 78).

**Characteristic**	**Value**
Age, years	Mean ± SD: 57.3 ± 11.5 Median (IQR): 58 (48.3–65.0)
Sex	Male: 58 (74.4%) Female: 20 (25.6%)
Ethnicity	Malay: 60 (76.9%) Indian: 11 (14.1%) Chinese: 5 (6.4%) Other: 2 (2.6%)
Employment Status	Unemployed: 27 (34.6%) Employed: 22 (28.2%) Retired: 23 (29.5%) Homemaker: 6 (7.7%)
Gross Household Income (RM)	Median (IQR): RM1450 (0–3000) Mean ± SD: RM1696.8 ± 1676.8
Number of Medication Classes	Mean ± SD: 6.6 ± 1.2 Median (IQR): 7 (6–7)
Full GDMT	Yes: 50 (64.1%) No: 28 (35.9%)

All patients met the definition of polypharmacy, with five or more medication classes prescribed. The mean number of medication classes was 6.6 (SD ±1.2), and the median was 7 (IQR: 6–7). Fifty patients (64.1%) were on full guideline-directed medical therapy (GDMT), defined as the concurrent use of a RAAS inhibitor (ACEi, ARB, or ARNI), beta blocker, MRA, and SGLT2i. A total of 64 patients (82.1%) were prescribed an SGLT2 inhibitor, regardless of whether they met full GDMT criteria.

The distribution of medication classes is detailed in Table 2. Statins were the most frequently prescribed (77 patients, 98.7%), followed by beta blockers (74 patients, 94.9%) and MRAs (73 patients, 93.6%). Antiplatelet agents were used by 62 patients (79.5%), and loop diuretics by 35 (44.9%). Among RAAS-modifying agents, ARNIs were most commonly used (47 patients, 60.3%), while ACE inhibitors and ARBs were less frequently prescribed (6 patients, 7.7% and 10 patients, 12.8%, respectively), indicating a clinical preference for ARNI where possible.

**Table 2:** Frequency of prescribed medication classes among included patients (*N* = 78).

Medication class	*n*, %
ACE inhibitor (ACEi)	6 (7.7%)
Angiotensin receptor blocker (ARB)	10 (12.8%)
Angiotensin receptor–neprilysin inhibitor (ARNI)	47 (60.3%)
Beta blocker	74 (94.9%)
Mineralocorticoid receptor antagonist (MRA)	73 (93.6%)
Sodium-glucose cotransporter 2 inhibitor (SGLT2i)	64 (82.1%)
Loop diuretic	35 (44.9%)
Antiplatelet agent	62 (79.5%)
Statin	77 (98.7%)
Insulin	10 (12.8%)
Oral hypoglycemic agents (OHA)	46 (59.0%)
Anticoagulant	11 (14.1%)

Diabetes management was evident in a large portion of the cohort, with 46 patients (59.0%) receiving oral hypoglycemic agents and 10 (12.8%) on insulin. Anticoagulants were prescribed to 11 patients (14.1%).

### Patient-Reported outcomes

PROs were assessed using three validated instruments: MyMAAT-12, the TBQ, and the WHOQOL-BREF.

The median adherence score (MyMAAT-12, 12 items) was 47.0 (IQR: 40.0–53.0), indicating generally low adherence. Most patients reported consistent medication use and appointment attendance. Items measuring unintentional non-adherence, such as forgetting doses (Q7) and missing medication without reminders (Q9), and intentional non-adherence, such as reducing medication when feeling better (Q2) or perceiving it unnecessary (Q6), all received high scores, indicating that these behaviors were uncommon.

The median treatment burden score, based on the 15-item TBQ, was 39.0 (IQR: 24.0–58.0), indicating a moderate perceived burden. Patients most commonly reported challenges related to self-monitoring, complex medication schedules, and attending follow-up appointments. Items relating to financial strain, dietary modifications, and social disruption scored lower on average, although some variability was observed.

The median QOL score, measured using the 26-item WHOQOL-BREF, was 88.0 (IQR: 77.0–98.0), suggesting generally favorable perceptions of health and well-being. The highest-rated domains included life enjoyment, bodily appearance, and sense of safety, while energy levels, sleep quality, and work capacity were comparatively lower. Full item-level data for all three instruments are presented in Table 3.

**Table 3:** Median and interquartile range (IQR) for individual items assessed by the Malaysia medication adherence assessment tool (MyMAAT-12), treatment burden questionnaire (TBQ), and WHOQOL-BREF among included patients (*N* = 78).

Item	Median	IQR
**A. MyMAAT-12—medication adherence**		
**I frequently failed to take my medication in accordance with the doctor’s instructions.**	5.0	4.0–5.0
**I reduced my medication intake when I felt better.**	5.0	5.0–5.0
**I took my medication on alternate days or skipped doses.**	5.0	5.0–5.0
**I was often late for or missed pharmacy appointments for my medication supply.**	5.0	5.0–5.0
**I kept more medication than needed at home.**	5.0	4.0–5.0
**I intentionally did not take my medication because I felt it was unnecessary.**	5.0	4.0–5.0
**I frequently forgot to take my medication.**	5.0	4.0–5.0
**I regularly took less medication than prescribed because I was afraid of side effects.**	4.0	4.0–5.0
**I often missed taking my medication if no one reminded me.**	4.0	3.0–5.0
**I was uncertain about how much medication I should take each day.**	4.0	4.0–5.0
**I was unable to manage my medication intake properly.**	4.0	3.0–5.0
**I lacked motivation to take my medication unless encouraged by my family or loved ones.**	4.0	3.0–5.0
**B. Treatment Burden Questionnaire (TBQ)**		
**The taste, shape, or size of your tablets made them difficult to take.**	2.0	1.0–5.0
**The number of times you had to take your medication daily was burdensome.**	3.0	2.0–5.0
**Remembering to take your medication (e.g., when away from home, using pillboxes) required a lot of effort.**	3.0	1.0–6.0
**You needed to follow special precautions with your medication (e.g., take with food, avoid certain activities).**	2.0	0.0–5.0
**You had to undergo frequent lab tests or medical examinations.**	3.0	1.0–5.0
**Self-monitoring tasks (e.g., measuring blood pressure, glucose) were burdensome.**	3.0	2.0–5.0
**Attending doctor appointments was difficult for you.**	2.0	1.0–5.0
**Your relationship with healthcare providers caused emotional or practical difficulties.**	3.0	1.0–5.0
**You had to frequently reorganize your daily routine due to treatment schedules.**	2.0	1.0–5.0
**Administrative tasks (e.g., reimbursements, hospital paperwork) were difficult to manage.**	2.0	0.0–5.0
**Healthcare-related expenses created financial burden.**	2.0	0.0–5.0
**Dietary changes required by your condition or treatment were difficult to maintain.**	2.0	0.0–4.0
**Following recommendations for physical activity was burdensome.**	3.0	1.0–5.0
**Your treatment or condition negatively affected your relationships with others.**	2.0	0.0–5.0
**Constant reminders of your health condition (due to treatment) were distressing.**	3.0	1.0–5.0
**C. WHOQOL-BREF—Quality of Life**		
**How would you rate your quality of life?**	4.0	3.0–5.0
**How satisfied are you with your health?**	4.0	3.0–5.0
**To what extent does physical pain prevent you from doing what you need to do?**	4.0	3.0–4.0
**How much do you need medical treatment to function in your daily life?**	3.0	3.0–4.0
**How much do you enjoy life?**	4.0	3.0–5.0
**To what extent do you feel your life to be meaningful?**	4.0	3.0–4.0
**How well are you able to concentrate?**	3.0	2.0–4.0
**How safe do you feel in your daily life?**	4.0	3.0–4.0
**How healthy is your physical environment?**	4.0	3.0–4.0
**Do you have enough energy for everyday life?**	4.0	3.0–5.0
**Are you able to accept your bodily appearance?**	4.0	3.0–5.0
**Have you enough money to meet your needs?**	4.0	3.0–5.0
**How available to you is the information that you need in your day-to-day life?**	4.0	3.0–4.0
**To what extent do you have the opportunity for leisure activities?**	4.0	3.0–4.0
**How well are you able to get around?**	4.0	3.0–4.0
**How satisfied are you with your sleep?**	4.0	3.0–4.0
**How satisfied are you with your ability to perform daily living activities?**	3.0	2.0–4.0
**How satisfied are you with your capacity for work?**	4.0	3.0–4.0
**How satisfied are you with yourself?**	4.0	3.0–4.0
**How satisfied are you with your personal relationships?**	4.0	3.0–4.0
**How satisfied are you with your sex life?**	4.0	3.0–4.0
**How satisfied are you with the support you get from your friends?**	4.0	3.0–4.0
**How satisfied are you with the conditions of your living place?**	4.0	3.0–5.0

### Comparisons by treatment groups

Subgroup analyses were conducted to assess differences in patient-reported outcomes based on guideline-directed medical therapy (GDMT) status, SGLT2i use, and ARNI prescription.

Among patients who were on full GDMT (*n* = 50), no statistically significant differences were observed in comparison to those not on full GDMT (*n* = 28) across all outcomes. Median adherence scores were similar (47.5 vs. 46.0; *p* = 0.34), as were treatment burden scores (38.0 vs. 40.0; *p* = 0.67) and quality of life scores (89.0 vs. 85.0; *p* = 0.18).

However, differences emerged when comparing SGLT2i users (*n* = 64) to non-users (*n* = 14). SGLT2i users reported lower adherence (median: 45.0 vs. 51.0; *p* = 0.037) and lower quality of life (median: 85.0 vs. 94.0; *p* = 0.018). There was no significant difference in treatment burden (*p* = 0.96).

In terms of RAAS inhibition strategies, a comparison between ARNI users (*n* = 47) and non-ARNI users (*n* = 31) also revealed no significant differences in any patient-reported outcomes. Median adherence scores were 57.0 in the ARNI group and 56.0 in the non-ARNI group (*p* = 0.393). Treatment burden scores were slightly higher in the ARNI group (29.0 vs. 27.0; *p* = 0.379), and quality of life scores were similar (73.0 vs. 75.0; *p* = 0.810).

### Correlation with medication load

Spearman correlation analysis revealed a positive association between the number of prescribed medication classes and QOL score (*r* = 0.295, *p* = 0.009), indicating that patients receiving more comprehensive therapy tended to report better quality of life.

No significant correlations were found between medication load and:

- Adherence score (*r* = 0.046, *p* = 0.691),
- Treatment burden score (*r* = –0.065, *p* = 0.571).

## Discussion

This study provides a detailed examination of the associations between therapeutic intensity and PROs—including medication adherence, treatment burden, and quality of life—among HF patients receiving care at an MTAC in Malaysia. Our findings demonstrate that even in the context of polypharmacy and the use of advanced therapies such as ARNI and SGLT2 inhibitors, patients reported moderate levels of adherence, manageable treatment burden, and favorable perceptions of quality of life. Nevertheless, these observations describe associations between therapeutic intensity and patient-reported outcomes within a pharmacist-led care setting and should not be interpreted as evidence of causal effects. In addition, the absence of adjustment for key clinical variables introduces the possibility of confounding by indication. Patients receiving more intensive therapy may have greater disease severity at baseline, which could influence both treatment exposure and patient-reported outcomes.

It is important to note a potential ceiling effect observed in the MyMAAT-12 responses, where item-level scores were predominantly clustered at higher Likert values (4–5). This pattern may reflect positive self-reporting tendencies or social desirability bias. However, the total adherence score incorporates multiple items, including those capturing less optimal behaviours, which may account for the overall median score falling below the predefined adherence threshold. This apparent discrepancy highlights the need for cautious interpretation of adherence findings in this study. As such, adherence levels may be overestimated at the item level, and the scale’s discriminative ability in this population may be limited, potentially reducing its sensitivity to detect meaningful differences between subgroups.

This limitation may also have attenuated observed associations between therapeutic intensity and adherence, and therefore the absence of significant differences across treatment groups should be interpreted with caution.

Polypharmacy is traditionally viewed as a risk factor for poor adherence, adverse drug events, and patient dissatisfaction, particularly in older adults and those with multimorbidity (10). However, in our cohort, which had a median of seven medication classes per patient, we observed no negative correlation between medication count and adherence or treatment burden. In fact, a weak-to-moderate positive association between medication load and quality of life was observed (Spearman's r = 0.295), explaining less than 10% of the variance. The underlying reasons for this association remain unclear and should be interpreted cautiously. Patients prescribed a higher number of medications may represent those receiving more intensive management due to greater disease burden, introducing the possibility of confounding by indication. As such, this observed association may reflect underlying differences in patient characteristics rather than a direct effect of medication load. The relationship is modest in magnitude and should therefore be interpreted cautiously. Nevertheless, even modest associations may be of interest in complex, multifactorial conditions such as heart failure.

This finding challenges the assumption that more medications inevitably equate to greater burden. It supports recent evidence suggesting that when medications are perceived as necessary, beneficial, and well-explained, patients are more likely to engage with and adhere to their regimens (20, 21). The MTAC model, by offering regular follow-up, individualized education, and medication reconciliation, may contribute to how patients interpret and manage polypharmacy from a burden into a managed necessity (19).

GDMT has been proven to reduce morbidity and mortality in HFrEF (2–9). However, real-world implementation of full GDMT remains suboptimal, often due to perceived concerns about patient tolerance, adherence, or polypharmacy burden (22, 23). In our study, full GDMT was prescribed in 64.1% of patients, and notably, no negative impact on any PRO domain was observed. Patients receiving full GDMT had adherence, treatment burden, and quality of life scores comparable to those not on full therapy. Similarly, ARNI users (60.3% of the cohort) had equivalent outcomes to non-users, suggesting that newer, evidence-based therapies do not necessarily compromise patient experience when delivered with adequate support. These findings suggest that comprehensive heart failure regimens are feasible and acceptable in this setting, particularly when paired with ongoing pharmacist engagement. These findings may help address concerns among clinicians regarding potential negative impacts of treatment escalation that may lead to poor adherence or dissatisfaction.

While the majority of patients (82.1%) were on SGLT2 inhibitors—reflecting good uptake of contemporary therapy—SGLT2 inhibitor use was associated with lower adherence and quality of life. These findings stand in contrast to their well-established clinical benefits in improving cardiovascular outcomes, reducing HF hospitalizations, and preserving renal function (8, 9, 13, 14). This observed association may reflect underlying differences in disease severity or clinical complexity rather than a direct effect of SGLT2 inhibitor therapy. The reasons behind this observation are likely multifactorial; however, these explanations remain speculative, as data on side effects, cost, and treatment duration were not collected in this study. SGLT2 inhibitors, though beneficial, are associated with side effects such as polyuria, dehydration, genital infections, and initial diuretic effects that may be distressing to patients (24). Additionally, in the Malaysian public healthcare context, issues related to access, out-of-pocket cost, and medication supply limitations may influence adherence and treatment perception. Patients newly initiated on SGLT2i may also experience transient discomfort or uncertainty, which could lower their self-rated quality of life. It is also possible that clinicians preferentially initiate SGLT2i in patients perceived to be at higher clinical risk or with more symptomatic disease, which could confound self-reported outcomes (25). These findings should be interpreted cautiously, as patients receiving SGLT2 inhibitors may represent a subgroup with greater disease severity or symptom burden. Although we did not assess HF severity or baseline NYHA class, such factors may underlie some of the observed differences and warrant further prospective investigation. Nevertheless, these hypotheses should be interpreted cautiously given the absence of direct data on these factors. In addition, the relatively small size of the non-SGLT2 inhibitor group may have limited statistical power and contributed to unstable estimates, increasing the risk of both type I and type II errors; therefore, these subgroup findings should be considered exploratory and hypothesis-generating. In particular, confounding by indication cannot be excluded, as patients initiated on SGLT2 inhibitors may represent a subgroup with more advanced or symptomatic disease.

Importantly, the observed associations between therapeutic intensity and patient-reported outcomes are likely influenced by underlying disease severity and may reflect confounding by indication. Patients with more advanced heart failure are more likely to receive intensified therapy, including full guideline-directed medical therapy and SGLT2 inhibitors, while simultaneously experiencing poorer quality of life and lower adherence. As such, the associations observed in this study should be interpreted with caution, as they may reflect differences in baseline clinical status rather than treatment effects.

The median TBQ score of 39.0 in our population indicates a moderate level of perceived treatment burden. This aligns with previous studies showing that patients with chronic conditions experience the greatest burden from frequent appointments, complex regimens, self-monitoring tasks, and administrative demands (26, 27). Interestingly, financial and social burdens were rated lower in our study, possibly reflecting the supportive nature of Malaysia's public health system and MTAC interventions that mitigate out-of-pocket expenses and psychosocial isolation. Notably, household income in this cohort showed considerable variability, with a substantial proportion of patients in lower-income brackets. Although cost-related data were not directly assessed, socioeconomic differences may influence medication adherence and perceived treatment burden, particularly with newer therapies such as SGLT2 inhibitors, and should be interpreted as a potential contributing factor. Given that treatment burden is increasingly recognized as a predictor of adherence and clinical outcomes (11), the relatively manageable burden scores in this cohort are consistent with a potential role of the MTAC model in helping patients manage complex treatment demands.

These findings have several practical implications. First, polypharmacy alone may not necessarily deter clinicians from pursuing optimal HF therapy, particularly in settings where pharmacist support is available. Second, targeted strategies are needed to support patients initiated on SGLT2 inhibitors, including enhanced counselling about side effects, expectations, and the long-term benefits of therapy. Third, the use of validated PRO tools such as MyMAAT-12, TBQ, and WHOQOL-BREF in routine care could offer valuable feedback loops for clinicians to identify and address patient challenges early. The results also underscore the need for personalized care models that balance evidence-based pharmacology with individual perceptions and capacity. MTAC services, by offering structured, multidisciplinary follow-up, may be well-positioned to support this balance.

Key strengths of this study include the use of locally validated, comprehensive patient-reported outcome instruments, a well-characterized real-world cohort, and the focus on a pharmacist-led care model that is central to national chronic disease strategies. However, several limitations should be acknowledged. The cross-sectional design precludes causal inference. The analyses were primarily univariable, and therefore the observed findings should not be interpreted as independent associations. Multivariable analyses were not performed due to sample size constraints, which may increase the risk of residual confounding and limit adjustment for potential clinical and sociodemographic factors. As such, the results remain susceptible to confounding by both measured and unmeasured variables. Future studies with larger sample sizes should incorporate multivariable modelling. As multivariable analyses were not performed, the observed associations do not represent independent effects and remain susceptible to confounding by both measured and unmeasured variables. In addition, important clinical markers of heart failure severity, including left ventricular ejection fraction, New York Heart Association class, NT-proBNP levels, renal function, and recent hospitalization status, were not available. The absence of these variables limits the ability to account for disease severity and may influence the observed associations. In addition, patient-reported outcomes are inherently subjective and may be influenced by recall or social desirability bias. Finally, as this was a single-center study with a predominantly Malay cohort, the findings may not be generalizable to other populations, healthcare systems, or settings without structured pharmacist-led services.

## Conclusion

In this real-world, cross-sectional study of heart failure patients managed in a pharmacist-led MTAC, therapeutic intensity, including polypharmacy, full guideline-directed medical therapy, and ARNI use, was not associated with increased treatment burden or reduced medication adherence and quality of life. A modest positive association between medication count and quality of life was observed; however, this finding should be interpreted cautiously, as it may reflect underlying differences in patient characteristics and disease severity rather than a direct effect of treatment.

SGLT2 inhibitor use was associated with lower adherence and quality of life, although these findings are exploratory and may be influenced by confounding by indication and treatment complexity.

Overall, these results provide observational insights into the relationship between therapeutic intensity and patient-reported outcomes within a pharmacist-led care setting and require confirmation in larger, longitudinal studies with appropriate adjustment for clinical variables.

## Data Availability

The raw data supporting the conclusions of this article will be made available by the authors, without undue reservation.
